# Quantitative evaluation of the human vocal fold extracellular matrix using multiphoton microscopy and optical coherence tomography

**DOI:** 10.1038/s41598-021-82157-9

**Published:** 2021-01-28

**Authors:** Fouzi Benboujja, Christopher Hartnick

**Affiliations:** Department of Otolaryngology, Massachusetts Eye and Ear Infirmary, Harvard Medical School, 243 Charles Street, Boston, MA 02114 USA

**Keywords:** Translational research, Oral anatomy, Biomedical engineering, Pathogenesis, Biophotonics

## Abstract

Identifying distinct normal extracellular matrix (ECM) features from pathology is of the upmost clinical importance for laryngeal diagnostics and therapy. Despite remarkable histological contributions, our understanding of the vocal fold (VF) physiology remains murky. The emerging field of non-invasive 3D optical imaging may be well-suited to unravel the complexity of the VF microanatomy. This study focused on characterizing the entire VF ECM in length and depth with optical imaging. A quantitative morphometric evaluation of the human vocal fold lamina propria using two-photon excitation fluorescence (TPEF), second harmonic generation (SHG), and optical coherence tomography (OCT) was investigated. Fibrillar morphological features, such as fiber diameter, orientation, anisotropy, waviness and second-order statistics features were evaluated and compared according to their spatial distribution. The evidence acquired in this study suggests that the VF ECM is not a strict discrete three-layer structure as traditionally described but instead a continuous assembly of different fibrillar arrangement anchored by predominant collagen transitions zones. We demonstrated that the ECM composition is distinct and markedly thinned in the anterior one-third of itself, which may play a role in the development of some laryngeal diseases. We further examined and extracted the relationship between OCT and multiphoton imaging, promoting correspondences that could lead to accurate 3D mapping of the VF architecture in real-time during phonosurgeries. As miniaturization of optical probes is consistently improving, a clinical translation of OCT imaging and multiphoton imaging, with valuable qualitative and quantitative features, may have significant implications for treating voice disorders.

## Introduction

**Figure 1:**
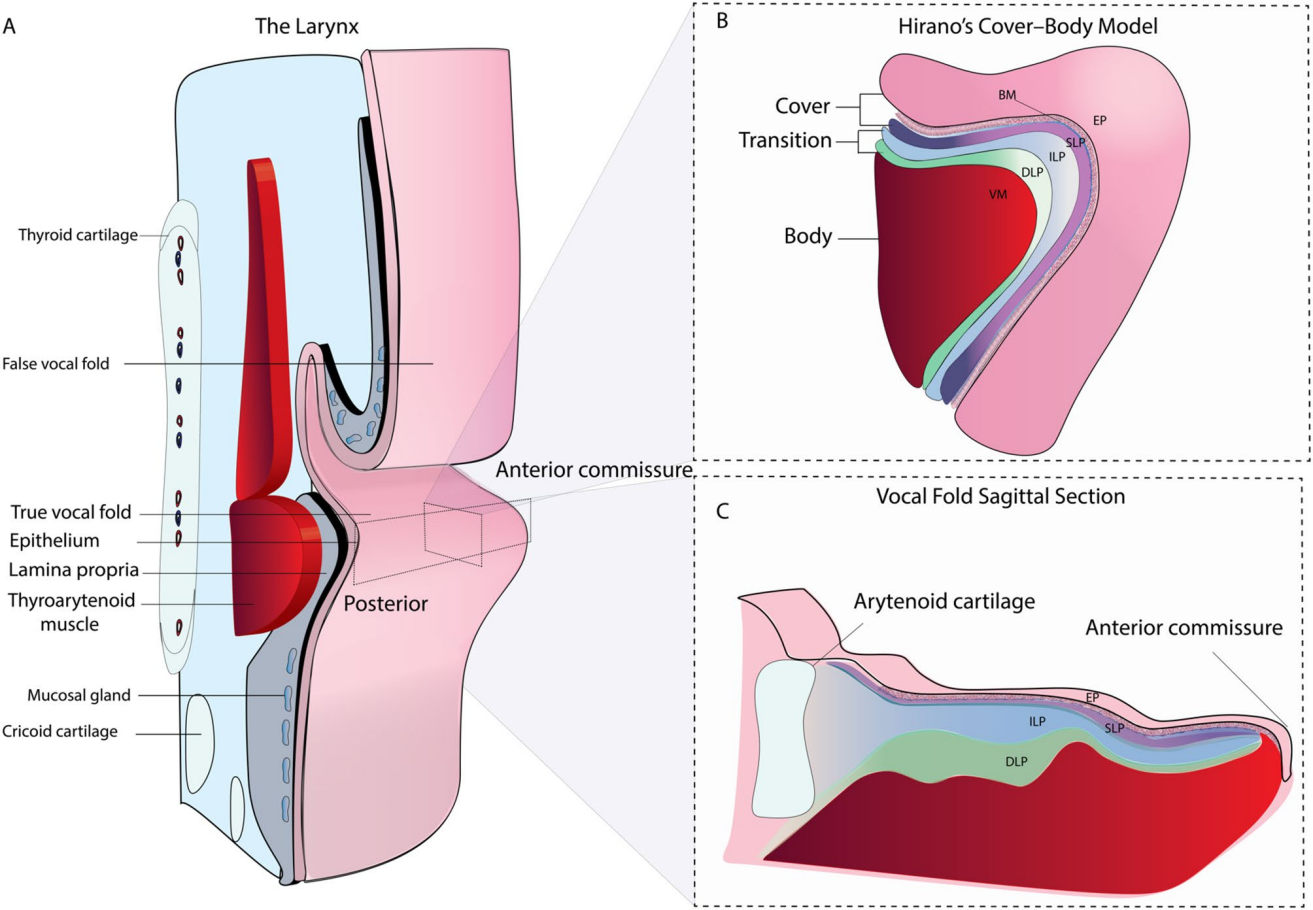
The human larynx. Major anatomical structures of the human larynx representing a complex organization of cartilages, ligaments, and muscles **(A)**. Attached anteriorly to the thyroid cartilage and posteriorly to the arytenoid cartilages, the true vocal folds are essential to phonation. Their physiology can be described with the cover-body theory of phonation **(B)**, which groups the epithelium (EP), the basement membrane (BM), and the superficial lamina propria (SLP) as a single entity: the cover, known to be critical for sustaining vibration. The vocal ligament is the transition layer between the cover and body, which include the intermediate lamina propria (ILP) and deep lamina propria (DLP). The body is represented by the thyroarytenoid (TA) muscle. Sagittal sections (anterior to posterior) used in this study allow for a full representation of the VF layered structure **(C)**.

The human larynx produces a staggering array of vibrational modes during phonation. This ability is provided in part by the vocal folds (VFs), a unique paired anatomical structure that plays an intricate role in voice production. Each VF consists of a very soft laminated structure of connective tissue, known as the lamina propria (LP) that sits between the overlying epithelium surface and the underlying thyroarytenoid muscle (Fig. 1A).

Our current understanding of the VF physiology and, therefore, how we treat the larynx is credited to Hirano’s mucoviscoelastic-aerodynamic model, also referred to as the cover-body theory of phonation^1^. Mainly, as illustrated in Fig. 1B, the epithelium (EP), the basement membrane (BM), and the superficial lamina propria (SLP), also known as the Reinke’s space, are grouped as a single entity: the cover, which is defined as critical for sustaining vibration during phonation^1,2^. The body is represented by the thyroarytenoid (TA) muscle which, sets in motion the vocal ligament i.e. the intermediate (ILP) and deep lamina propria (DLP). The vocal ligament is the transition layer between the cover and body^2,3^. These LP subdivisions are based on histological observations of the extracellular matrix (ECM) fibrillar composition. Thus, it is generally accepted that elastin and collagen fibrous macromolecules and interstitial proteins are fundamental to pliability and extensibility properties of the VF^4,5^. However, pathology, surgery, and treatments easily alter ECM homeostasis, thus disrupting the normal vibratory function, and thereby affecting the ability to modulate airflow. Moreover, discriminating between benign and malignant lesions is mainly biopsy driven and continues to be a diagnostic challenge endangering patient with vocal scars and permanent dysphonia.

Therefore, microlaryngeal techniques have been developed to preserve, to a maximum extent, the microanatomy of the VF, and its composition has been extensively studied with histopathology analysis^1,2,6–9^ and scanning electron microscopy (SEM)^10,11^. Despite these efforts, it remains challenging to extend such two-dimensional histological investigations to the patient's structure-functional anatomy. Consequently, 3D imaging modalities such as ultrasound (US)^12^, computed tomography (CT)^13,14^, and magnetic resonance imaging (MRI)^15^ have been investigated on the VF mucosa; however, their findings all report the insufficient spatial resolution to visualize micron-scale structures of the ECM.

Recently, synchrotron X-ray microtomography have been used successfully on human cadavers^16^, and nanotomography on rabbits^17^ to identify the tri-dimensional structures of the VF ECM, including its muscle fibers morphology. However, these techniques are not collagen or elastin sensitive and suffer from low contrast since they instead rely on tissue density (X-ray attenuation coefficient) as a contrast mechanism. Although these methods certainly provide valuable insight into the human VFs, their availability is extremely scarce, with technical challenges that hinder their extensive application. Furthermore, similarly to SEM, these techniques are labor-intensive and detrimental to the tissue, which precludes their clinical application for in vivo imaging guidance.

The emerging field of optical imaging allows a transition from a 2D human larynx to a 3D detailed description of the VF. Among non-invasive imaging techniques, optical coherence tomography (OCT)^18^ and multiphoton microscopy^19^ are attractive modalities with sufficient resolution and contrast to identify and track patient’s anatomy. Additionally, both modalities can image thick samples without any biopsy, sample preparation, or tissue processing (labeling staining methods).

OCT is a non-invasive and highly sensitive optical imaging modality. Through an interferometric scheme, ballistic photons (and quasi-ballistic photons) from a broadband source are backscattered by the tissue and detected^18^. The short temporal coherence of the light source, combined to a low attenuation of biological tissue under near-infrared wavelengths (ranging from 800 to 1300 nm), enables depth-resolved images up to 2.5 mm with a high axial resolution (1 to 12 μm)^20–23^. Its clinical potential has been well-demonstrated in laryngeal studies, such as in subglottic stenosis in adults^24–26^, and neonates^27^, vibrating VFs^28–30^, VF injections^31^ and with benign and malignant lesions^24,28,30,32–36^. Furthermore, OCT provides means to characterize and quantify the mucosa motion with doppler analysis^28–30^, describe subglottic injuries with a second-order statistics^37^, and its composition with attenuation coefficients^38,39^. Its main limitation pertains to the clinical interpretation of OCT VF images (optical scattering).

Among multiphoton microscopy techniques, two-photon excitation fluorescence microscopy (TFEM) and second harmonic generation (SHG) imaging are well-suited to investigate the VF ECM. TFEM enables the identification of endogenous fluorophores such as nicotinamide adenine dinucleotide phosphate (NAD(P)H), flavoproteins, elastin, and hemoglobin^40–42^, while SHG has shown to be useful for investigating ordered structural protein such as collagen fibers in intact tissue^43^. Both signals can be acquired simultaneously, using a single excitation wavelength, providing a powerful multi-contrast approach. Although ex vivo human VFs^44–47^ have been imaged under multiphoton microscopy, only a small dissected volume, covering 10% of the total VF length was investigated. The bulky setup and scanning speed are the shortcomings of multiphoton microscopy. However, recent developments have been proposed to transition from a bench to a real-time handheld rigid multiphoton endoscope compatible with laryngeal requirements (length > 15 mm and outside diameter < 2.7 mm)^48^.

Despite the remarkable contribution of histological findings and other imaging modalities to our understanding of the VF physiology, the relationship between the LP layered properties and pathology remains murky. A complete picture of its functional anatomy requires a quantitative assessment on the VF ECM on its entire length, from the arytenoid cartilage to the anterior commissure, as shown in Fig. 1C. This study shows the first quantitative analysis of the entire VF ECM using multiphoton microscopy. Furthermore, side-by-side comparison with OCT was never investigated, which could be of potential value for harnessing both modality strengths (molecular contrast/ in-depth imaging with fast volume acquisitions) for laryngeal diagnostics and therapy.

As miniaturization of optical probes are consistently improving, a translation of OCT imaging and multiphoton imaging to the human larynx may have significant implications for treating voice disorders efficiently as a function of age and gender. In addition to investigating the potential for those modalities to assess and characterize the VF ECM, this study is further motivated by determining if non-invasive optical imaging modalities can address fundamental questions about the normal VF microanatomy.

1. Is the discrete three-layer structure visible under label-free imaging such as two-photon excitation fluorescence microscopy, second harmonic generation (SHG) imaging, and OCT?
2. Is there any variations in the ECM along the VF length (anteroposterior axis)?
3. Are they distinct features of the ECM that predisposes vocal lesions such as VF nodules in the anterior third?
4. Are they optical features that may accurately identify normal from pathology and guide phonosurgeries?

## Materials and methods

### Study design

Our investigation consists of imaging the subcellular morphology of the human VF with two-photon excitation fluorescence (TPEF), second harmonic generation (SHG) imaging, and optical coherence tomography (OCT). Morphometrics and fibrillar spatial distributions are compared between modalities and validated with histopathology. Human larynges were obtained from the Massachusetts General Hospital donor bank, along with written and informed consent. This study was approved by Massachusetts Eye and Ear Infirmary Institutional Review Board in accordance with research regulations, guidelines, and ethical protocols established by the Mass General Brigham.

### Tissue specimen preparation

**Figure 2:**
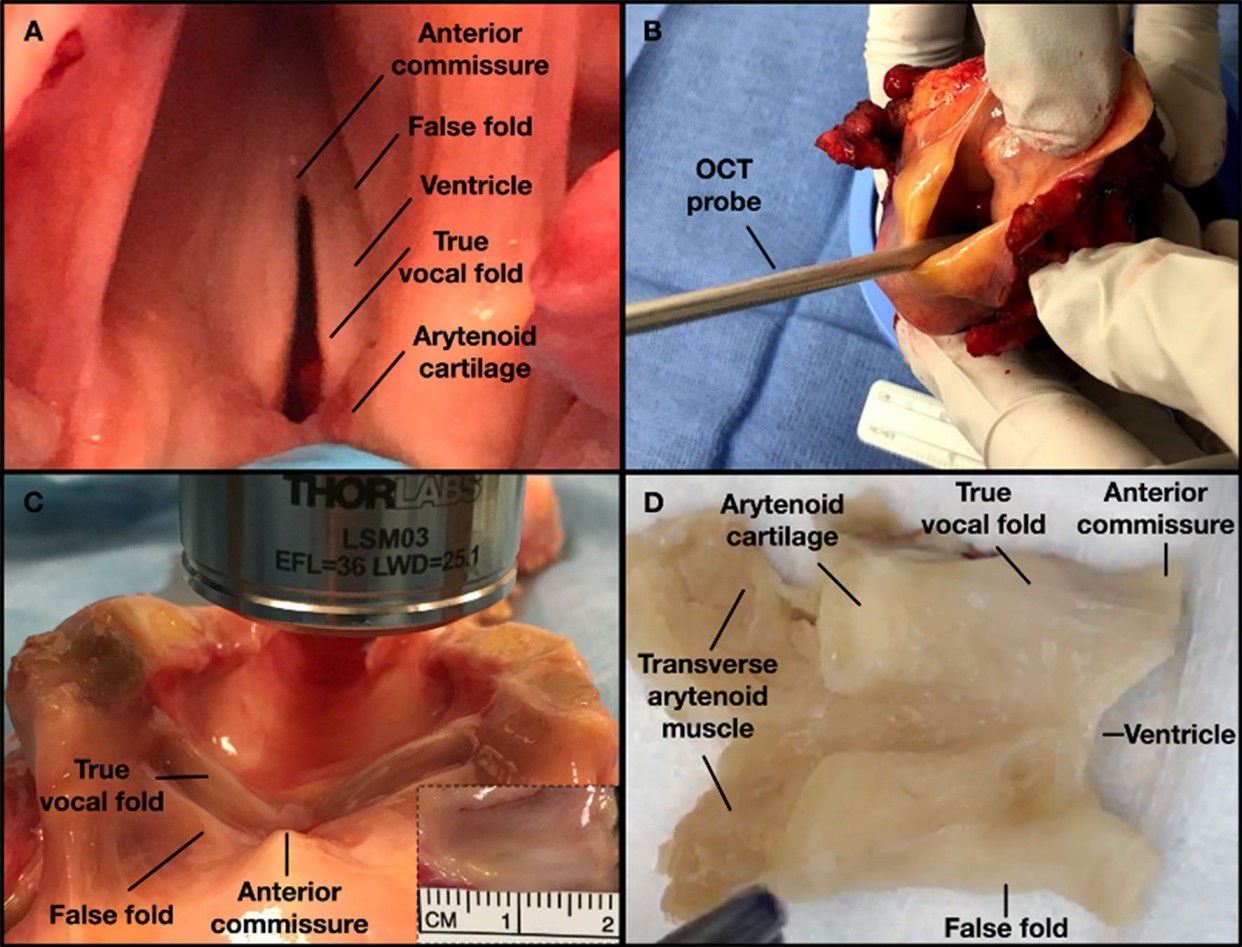
Experimental protocol used to characterize the vocal fold. A 66-year-old male larynx with vocal folds showing no signs of trauma **(A)**. The larynx was imaged under OCT with a handheld probe **(B)** and a bench-top system **(C)** for a wider field-of-view. Inset representing length measurements acquired after dissection of true and false vocal fold. Hemi-laryngeal structures (**D**) prior sectioning and after a neutral formalin immersion.

We examined two cadaveric larynges (66-year-old male and one 50-year-old female) obtained from subjects with no history of intubation, laryngeal diseases, dysphonia, or trauma. After a gross inspection (Fig. 2A), the harvested larynges were stored immediately in a phosphate-buffered saline solution at 4 °C. The intact larynges were first imaged with an OCT endoscopic probe (Fig. 2B) followed by an OCT bench-top system for a broader perspective, after opening the larynx posteriorly (Fig. 2C). The measured length of the VFs was 14 and 17 mm for the female and male, respectively (inset Fig. 2C). After imaging, the VFs were immediately retained in a 10% neutral buffered formalin solution, and further paraffin-embedded processed for sectioning (Fig. 2D).

### OCT imaging

The tip of a custom handheld OCT probe, previously described^33^, was placed carefully on the true VF at normal resting strain and was manually swept along the anteroposterior axis, and further swept inferiorly to superiorly along the mid-membranous portion of the VF. The handheld probe is built on a grin-lens relay enclosed with a thin endoscopic protective sheath (Slide-ON Endosheath, Medtronic) for an overall 3.6 mm in diameter. The lateral raster scan is implemented with two mirror-mounted galvanometers (6215H, Cambridge Technology) and enables a 2 mm^2^ lateral field of view (FOV). The probe is coupled to a tunable laser light source (SL1310V1, Thorlabs) centered at 1300 nm with a tuning range of 110 nm (at full width at half maximum) with a sweep repetition rate of 100 kHz and average output power of 40 mW (5 mW on the tissue). The axial and lateral resolutions are 12 and 25 microns in tissue, respectively. The maximum sensitivity of the system was measured to be 108 dB. Following the endoscopic scan, the larynx was opened posteriorly, and the thyroid cartilage was pinned to the table to allow a second OCT acquisition to be acquired on a benchtop system with a commercial objective lens (LSM03, Thorlabs) with a 36 mm effective focal length, and a 9.3 mm^2^ field of view. The benchtop system measured lateral resolution was 25 microns and allowed to capture a three-dimensional volume (10 mm, 5 mm, 5 mm, 1040 × 1040 × 1040 voxels) in a single scan without any manual sweep. The data was acquired with a ThorImage (version 4.4, Thorlabs) and process with Fiji^49^ (version 2.0).

### Histochemical evaluation

Multiple consecutive five-micron sagittal sections were acquired, which includes the anterior commissure, the transverse arytenoid muscle, the false and true VF. Sections were stained with Hematoxylin and eosin (nuclei, extracellular matrix, and cytoplasm), Masson trichrome (collagen fibers, smooth muscles), Verhoeff (mature elastic fibers) and Picrosirius red (collagen birefringence, muscle fibers, and cytoplasm) to enhance the visualization of specific structures. Additionally, every fifth slide, a 20-micron section was acquired and was left unstained for multiphoton imaging. This sectioning-staining protocol was reiterated multiple times throughout the sample along the medial to lateral direction. Histochemical microscopy was performed across different regions of the lamina propria detailing the microstructure under multiple magnifications. Each slide was imaged with a widefield microscope (BZ-X800, Keyence) under a 10x, 20 × and 60 × objective (CFI Plan Apo, NA 0.45, CFI Plan Fluor, NA 0.75, CFI Plan Apo, NA 0.95, Nikon). Brightfield and fluorescence images captured were automatically stitched with Analysis (version 2.1, Keyence).

### Multiphoton imaging

Individual and consecutive unstained slides of 20 μm were imaged under a state-of-the-art customized two-photon system (TrimScope II, LaVision Biotech) with galvanometers and resonant scanners capable of imaging depths up to half-millimeter at video rate. TPEF and SHG signals were collected using a tunable femtosecond laser (InSight X3, Spectra-Physics) that emits in the range of 680 to 1300 nm. All our acquisitions were set to a 920 nm excitation wavelength. The SHG signal showed the best signal to noise ratio under this wavelength, similar to previous skin collagen studies^43,50^. A dichroic mirror separates excitation and emission light (720 nm cut off). The emission light is further split by a dichroic mirror (495 nm long-pass filter). A full sweep was performed with a 20 × water immersion objective lens (NA 1.05, Zeiss). The tissue was scanned axially every 0.5 μm through a motor-driven XY-table with a Z-travel (Intravital, LaVision Biotech) that enable the collection of volumetric datasets. Autofluorescence and backward SHG signals were simultaneously collected through emission filters (Semrock 461/10 nm, 525/50 nm) and two GaAsP photomultiplier tubes (H7422-40/50, Hamamatsu). Each image covered a field of view of ∼440 × 440 μm (1024 × 1024 pixels) and was averaged four times to improve the signal to noise ratio. The entire human VF's subcellular morphology was reconstructed and stitched with TeraStitcher^51^.

### Quantitative analysis

**Figure 3:**
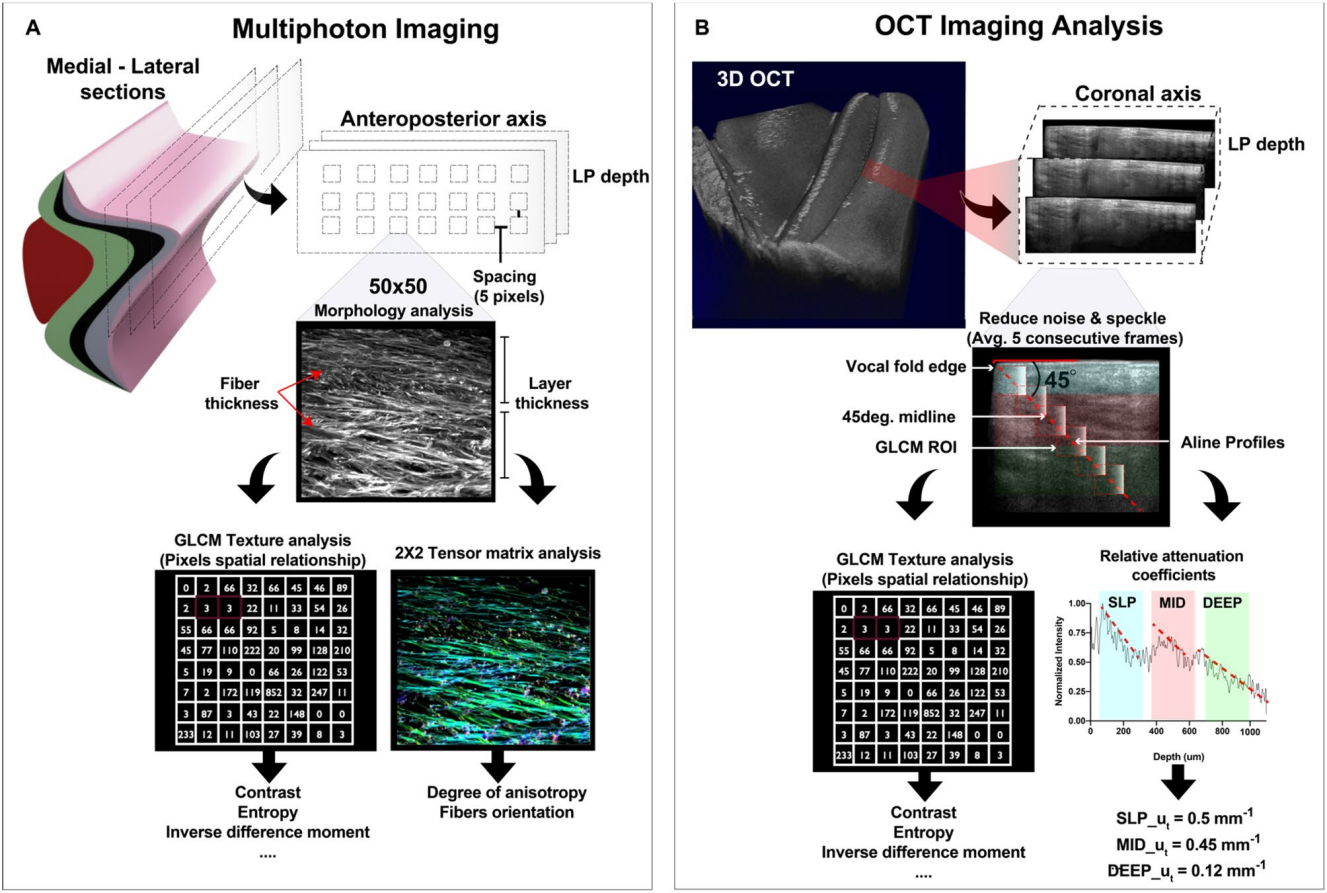
Multiphoton and OCT quantitative analysis. The anteroposterior sagittal sections were divided into multiple 50 × 50 × 50 pixels windows. Each window representing a position in the lamina propria (LP) was processed through a gray-level co-occurrence matrix (GLCM) and a structure tensor **(A)**. Features extracted are the collagen and elastic fiber diameters, the degree of anisotropy, fiber principal orientation, and second-order statistics. OCT images were averaged to improve the signal to noise ratio prior to the texture and relative attenuation analysis **(B)**.

This study’s premise was to extract features between modalities to gain valuable insight into the VF's morphologic and functional microanatomy. As illustrated in Fig. 3, we analyzed structural morphological changes associated with collagen in different ECM regions by extracting optical features in multiphoton and OCT images. As a first step, a texture analysis was performed on both sets of images. Additionally, a structure tensor was applied on SHG images to extract the dominant direction of collagen fibers and the anisotropy for each relevant VF section (Fig. 3A). In addition, a relative attenuation coefficient analysis was performed on OCT images to characterize how the ECM attenuates light according to its depth (Fig. 3B).

#### Second-order statistics

Texture analysis was extensively used in OCT and SHG to characterize the human tissue^52–55^. GLCM is among the most frequently used statistical method to quantitatively distinguish the normal morphology from pathology. This study used the GLCM to extract collagen fibers’ spatial arrangement in SHG images and further analyze the speckle distribution in OCT images. The texture analysis estimates the probability of a gray-tone occurrence between a pair of pixels $(i,j)$, separated by distance $d$ and a direction $\theta$. This joint conditional probability density function ${P}_{d,\theta }(i,j)$ was computed for individual windows of 50 × 50 pixels on OCT and SHG raw images. A spacing of 10% was set (5 pixels) between individual windows in each direction to avoid an overlap in the analysis. The GLCM was computed for 1- and 2-pixels neighborhoods along 0°, 45°, 90°, and 135°. All four orientations around their respective neighborhoods were average to a single value that characterized that window at that specific depth. The algorithm was implemented around GLCM functions available in the MATLAB image processing toolbox. Out of the 14 standard GLCM features reported^56^, the contrast, entropy, and inverse difference moment (IDM) were analyzed more profoundly. These features are well-suited to represent collagen morphology^57^. Altogether, those properties quantify the degree of heterogeneity (contrast), homogeneity (IDM), and randomness (entropy).

#### Orientation and anisotropy

Although the fast Fourier transform (FFT) is the most applied technique to extract quantitative spatial metrics for elastin and collagen fibers^58,59^, structure tensors have shown impressive outcomes for extracting cardiovascular tissue^60–62^, cornea collagen networks^63^ and nerve connectivity in the human brain^64^. This study used structure tensors to extract the vocal fold collagen predominant fiber orientations and its degree of anisotropy. The structure tensor is a second-moment matrix computed from the image gradients. Implemented in MATLAB, 16-bit SHG raw images were converted into 8-bit grayscale images, where 0 and 255 correspond to black and white pixel, respectively. Prior to constructing the matrix, SHG images are divided into small individual windows of 50 × 50 pixels. Each individual window is convoluted with a Gaussian matrix ${K}_{\sigma }$ with a kernel size $\sigma$ = 2 pixels to smooth the data. This gaussian kernel was selected to roughly match the collagen fibers diameter, which varies between 1 and 3 pixels (1–5 um) according to the region of interest (SLP, SVLT, MID, IVLT, DEEP). Following the smoothing step, the Euclidean distance (norm) was calculated with the partial derivatives along x and y directions for each individual window. The partial derivatives were computed through a cubic spline interpolation^65^ to form the final structure tensor ${S}_{T}$.

1 $${S}_{T}=\left(\begin{array}{cc}{{K}_{\sigma }*SHG}_{x}^{2}& {K}_{\sigma }*SH{G}_{xy}\\ {K}_{\sigma }*SH{G}_{yx}& {K}_{\sigma }*{SHG}_{y}^{2}\end{array}\right)$$

After generating the second-moment matrix ${S}_{T}$, the preferential orientation $\theta$ was extracted^66^.

2 $$\theta = \frac{1}{2}\mathrm{arctan}\left(\frac{2{(K}_{\sigma }*SH{G}_{xy})}{{({K}_{\sigma }*SHG}_{y}^{2})- {{(K}_{\sigma }*SHG}_{x}^{2})}\right)$$

$\theta$ is independent of sample position and represents the relative angle formed by collagen fibers and the epithelium surface, with positive angles measured counterclockwise. For instance, it is well known that many collagen fibers run from the anterior to posterior VF, parallel to the surface; those fibers are oriented and labeled as $\theta$ = 0$^\circ$.

The eigenvalues ($${\lambda }_{1}$$, $${\lambda }_{2}$$) of $${S}_{T}$$ carry the information about the distribution of the gradient and are extracted along with the eigenvectors ($${{e}_{1},e}_{2})$$. Roughly equivalent eigenvalues $$\left|{\lambda }_{1}\right|$$ $$\approx \left|{\lambda }_{2}\right|$$ are considered to represent a homogenous window. Otherwise, the predominant orientation lies with or in between the gradient directions. The coherence $$(\mathrm{C})$$, which indicates the disparity between eigenvalues ($${\lambda }_{\mathrm{max}}$$, $${\lambda }_{\mathrm{min}}$$) or the confidence towards rotational symmetry, was computed^66,67^ and labeled as the anisotropy ($$A).$$

3 $$A=\mathrm{C }=\left(\frac{{\lambda }_{\mathrm{max}}- {\lambda }_{\mathrm{min}}}{{\lambda }_{\mathrm{max}}+ {\lambda }_{min}}\right) $$

The extracted degree of anisotropy is dimensionless and varies between 0 and 1. It is maximum when collagen fibers are oriented in one direction and null if the images show an isotropic distribution. Fifty measurements for a region of interest were averaged to obtain the orientation and the standard deviation representing the region under investigation (SLP, SVLT, MID, IVLT, DEEP).

#### Relative attenuation analysis on OCT images

Attenuation in OCT is an important parameter that characterizes how quickly the signal decays due to tissue absorption and scattering. This relationship is governed by the Beer-Lambert law, where

4 $$\langle I(z)\rangle  \propto {I}_{0} \mathrm{exp}(-2{u}_{t}\cdot z)$$

the average OCT signal $$\langle I\left(z\right)\rangle $$ detected is proportional to the reflected intensity resulting from the scatters present in the tissue (cell membranes, collagen and elastic fibers) at different depth ($$z)$$. $${I}_{0}$$ is the initial OCT signal amplitude at the epithelium surface before interacting with tissue ($$z=0$$). Although the tissue attenuation coefficient $${u}_{t}$$ is a combination of the absorption ($${u}_{a}$$) and scattering ($${u}_{s}$$) coefficients, near the infrared-regime (1300 nm) the signal is dominated mainly by backscattering properties. The factor 2 accounts for round trip attenuation. Attenuation coefficients were used successfully for differentiating atherosclerotic tissue^68,69^, detecting axillary lymph node cancer^70^, glaucoma^71^, and oral epithelial dysplasia^72^. In this study, as illustrated in Fig. 3B, before computing the relative attenuation coefficients $${u}_{t}$$ for each region of interest of the VF, the speckle noise was reduced by averaging five consecutive frames, and the signal was normalized for all patients to account for environmental variation between acquisitions (power source, tissue viscosity). Implemented in MATLAB, and as previously described^73,74^, the relative attenuation coefficients were estimated through a linear regression analysis on averaged logarithm scaled intensity profiles (A-lines) according to the single-scattering model^75^. To reduce bias in the selection of the region of analysis across volumes, we extracted features along the 45° midline from the VF edge epithelium. The 45° landmark is easily identifiable and trackable during endoscopy^34^. It allows to symmetrically cross the vocal fold edge and all layers of the lamina propria. Furthermore, it is a region of clinical importance as the vocal fold edge is susceptible to mechanical stresses during phonation and, therefore, to pathology^76^. We adjusted OCT thickness values (using Pythagorean’s theorem) before comparison and validation with SHG measurements. The refractive index, which governs the pixel size, was set to n = 1.384^77^. This value correlates with histopathology morphometric studies^39,78^.

#### Morphometrics and comparison of measured features

The contrast available in high-resolution multiphoton images allows for observing and compiling features in five LP segments (SLP, SVLT, MID, IVLT, DEEP), while on coronal OCT images, only three segments could be differentiated and analyzed (SLP, MID, and DEEP). The intertwined mesh of collagen and elastic fibers visible under SHG presents natural morphologic features that differentiate each LP segment. Collagen fibers diameter, fiber densities (packed collagen fiber bundles), and the number of fenestration (void regions) were used to differentiate each ECM layer. The various fenestration (void regions) in ECM was evaluated by computing the proportion of void regions (black pixels) relative to regions with collagen (gray-tone pixels). The thickness for each LP segment was measured manually with the Fiji scale tool and was further validated with the relative attenuation analysis in OCT, where the length of distinctive attenuation profiles indicates the layer thickness. Moreover, the fibers’ diameter in SHG images, which predicated the structure tensor Gaussian kernel size, were also measured with Fiji. According to the LP segments, the computed optical and morphometrics features were evaluated with a one-way analysis of variance (ANOVA) in GraphPad Prism (version 8.4, GraphPad Software Inc). Pairwise differences were performed using Tukey's range test, and a value of P < 0.001 was considered extremely significant.

## Results

### Histochemical evaluation

**Figure 4:**
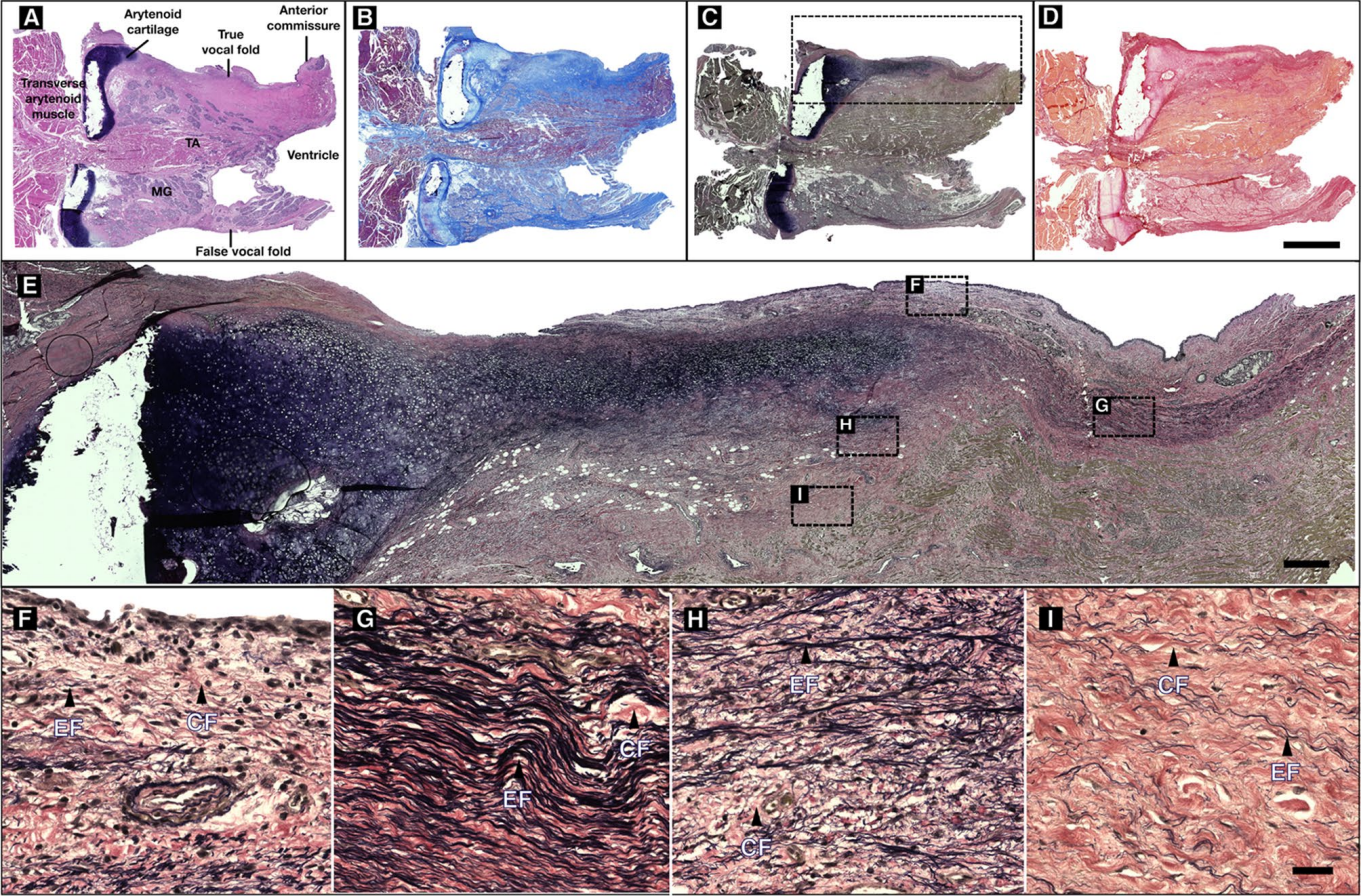
Sagittal sections of the human adult vocal fold. Medial **(A)** to lateral **(D)** sagittal sections of a 50-year-old female vocal fold under low magnification (10x). Each slide from the anterior commissure to the vocal process shows a different contrast. The true vocal fold thickens posteriorly and laterally. Verhoeff stain **(C)** highlight the elastic fibers band (stained in black) that extend along the full length of the vocal fold with inset showing a magnification at × 20 (E). The distribution of elastin varies according to the depth and anteroposterior position on the true vocal fold **(E)**, as shown through a × 60 magnification on insets **(F–I)** with arrows pointing to elastic (EF) and collagen fibers (CF). **(A)** Hematoxylin and eosin, **(B)** Masson trichome, **(C,F–I)** Verhoeff. **(D)** Picrosirius red – Scale bars: 5 mm [**A**–**D**], 500 μm [**E**], 50 μm [**F**–**I**].

To investigate the morphology and characteristic of the VF ECM consecutives five-micron sagittal sections were acquired on human larynges. Figure 4 shows representative sagittal sections of the human adult VF, from the medial (Fig. 4A) to the lateral aspect of the VF (Fig. 4D). Its entire length is visible at low magnification (10x), from the anterior commissure to the arytenoid cartilage, with the true and false ventricular fold parted by the thyroarytenoid (TA) muscle and the ventricle. Under all histological staining sections observed (Fig. 4A–D), the fiber distribution in the false VFs does not suggest any layered properties (i.e., variation in the fiber density).

In opposition to the false VF, the true VF mucosa is voided of any glands. Its shape and composition change along its length, suggesting a complex collagen and elastin distribution. For instance, the Verhoeff’s slides show a clear elastic band (stained in black) delimitating the superficial and the deep region (Fig. 4E). This band extends from the vocal process of the arytenoid cartilage to the anterior commissure and varies in density along the anteroposterior axis. At higher magnification (× 60), the arrangement, population density of elastic (stained in black), and collagen fibers (stained in pink) differs according to LP location (Fig. 4F–I).

Although wide-field microscopy highlights fibrous structures in the extracellular matrix it remains challenging to quantify the arrangement and orientation of collagen and elastic fibers without chemical processes to remove either elastin or collagen as both are intimately intertwined^79^. It should be emphasized that while a density gradient is visible in histological sections (Fig. 4A–D), its distribution and magnitude depend on the anteroposterior axis and the staining protocol.

### Multiphoton imaging

#### ECM qualitative analysis

**Figure 5:**
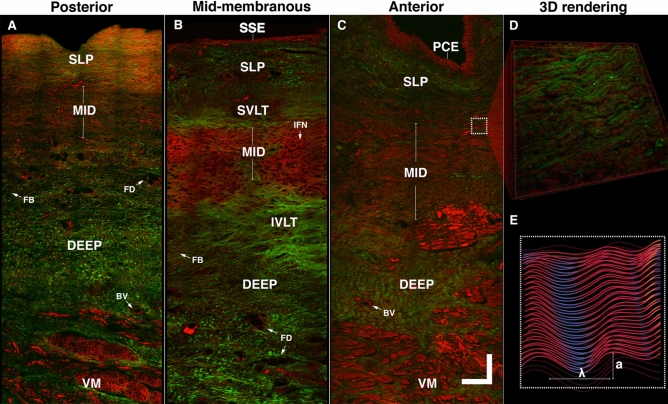
Characterization of the human adult vocal fold using two-photon excitation fluorescence (TPEF) and second harmonic generation (SHG). Composite image showing TPEF (color-coded red) and SHG (color-coded green) of a 50-year-old female vocal fold (VF) imaged with a 920 nm excitation wavelength. The posterior **(A)**, mid-membranous **(B)**, and anterior **(C)** section of the VF shows a different fiber distribution. A delicate intertwined fibrous network (IFN) is visible in the mid-membranous region. The depth and the anteroposterior position dictate the density and maturity of elastic and collagen fibers. The inset shows the 3D rendering of the fiber waviness in the anterior region **(D)** and fiber waviness schematic **(F)**. *SSE* stratified squamous epithelium; *PCE* pseudostratified ciliated epithelium; *SLP* superficial lamina propria; *MID* mid lamina propria; *Deep* deep lamina propria; *IFN* intertwined fiber network; *SVLT* superior vocal ligament transition; *IVLT* inferior vocal ligament transition; *BV* blood vessel; *FB* fibroblasts; *FD* fat droplets; *VM* vocalis muscle. Scalebars: 150 μm.

Multiphoton imaging is uniquely suited to analyze tissue and reconstructs 3D laryngeal anatomy with subcellular resolution without staining. Figure 5 shows a sagittal cross-section along the full-length of the true VF under TPEF (color-coded in red) and SHG (color-coded in green). Epithelial cells, collagen, and elastic fibers are segmented in three tiers, as it is often clinically referred to as anterior, mid-membranous, and posterior. It is noteworthy the striking difference in the optical contrast (label-free) from the three regions.

TPEF shows a stratified squamous non-keratinized epithelium (SSE) layer along the VF length except for the anterior region (Fig. 5C), which is occupied by pseudostratified ciliated cells (PCE). Beneath the epithelium, the anterior SLP shows predominantly collagen fibers (Fig. 5C), while the posterior SLP is dominated by elastic fibers (Fig. 5A). The mid-membranous SLP is distinct from both extremities and shows elastic and collagen fibers loosely arranged, representative of a hypocellular region (Fig. 5B). At mid-depth (MID), the anterior region is composed of mature elastic and collagen fibers, running parallel to the VF edge in opposition to a delicate intertwined fibrous network (IFN) in the mid-membranous region (Fig. 5B). A closer analysis reveals superior and inferior vocal ligament transitions (SVLT, IVLT). These zones are described as a 3D spatial arrangement of collagen fibers anchoring the mid-layer to the superficial and deep LP. Furthermore, mature elastic fibers are mostly distributed along the mid-region, with relatively lower concentrations in the deeper LP. This observation was also found in previous histological analyses^80^. Moreover, fat droplets (FD), blood vessels (BV), and numerous fibroblasts (FB) are visible deep in the LP, while collagen fibers are seen interlinked and anchored in the vocalis muscle (VM).

The 3D rendering shows elastin and collagen fibers in the anterior region, forming a quasi-sinusoidal pattern (Fig. 5D). The fiber waviness delimited by peaks and valleys is observed at different scales. Locally at the fiber scale, a spatial period of $\lambda =$ 18 μm (± 9 μm ) is visible, with an amplitude $a=$ 9 μm (± 5 μm ) however with a larger field of view fiber bundles also creates a global spatial pattern that reaches $\lambda =$ 97 μm (± 23 μm) peak to peak. It is worth noting that the waviness is not generalizable along the full length of the VF and remains firmly spatially dependent on the VF LP region.

#### ECM quantitative analysis

**Figure 6:**
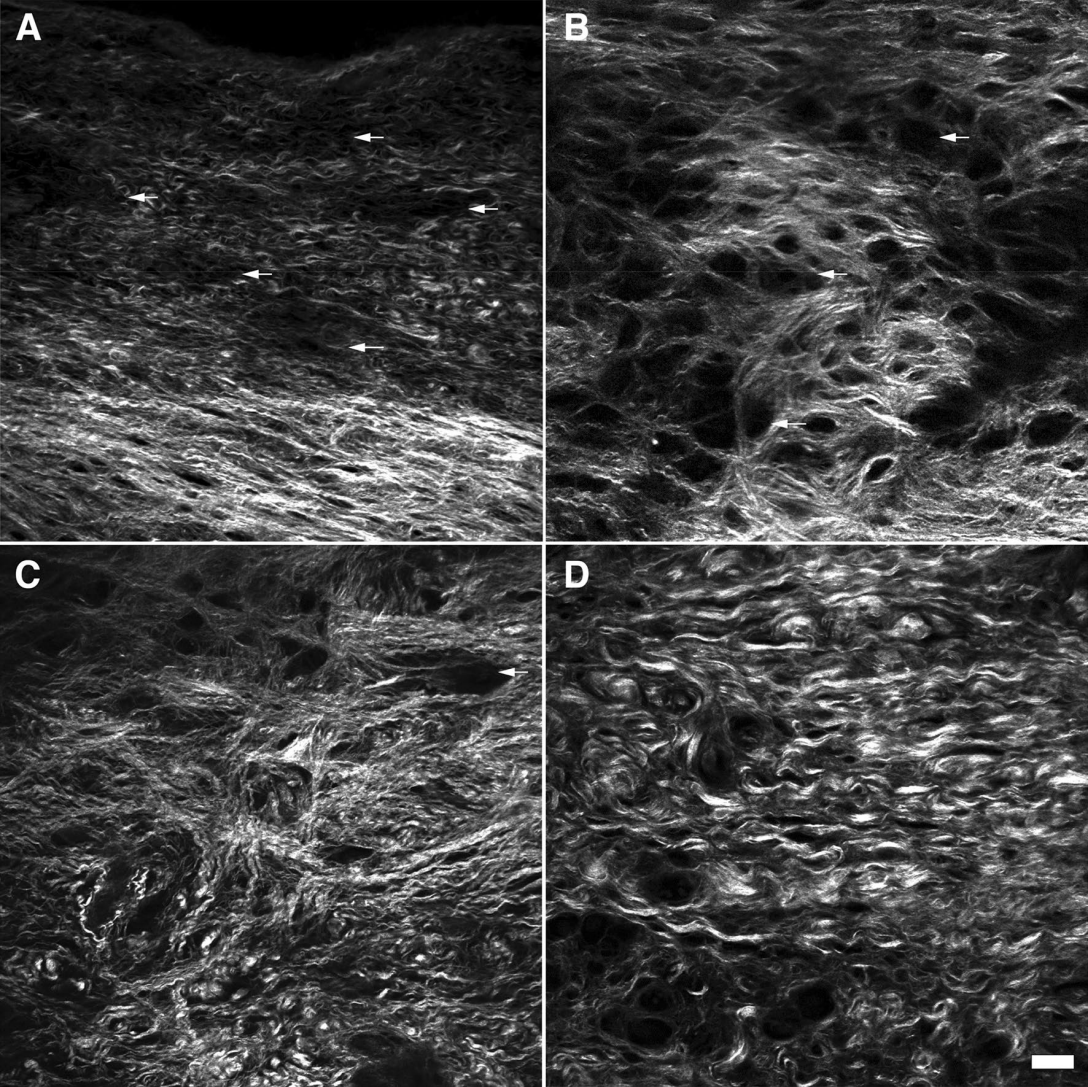
Shape and arrangement of collagen fibers in the mid-membranous adult vocal fold observed under SHG. A 50-year-old female vocal fold lamina propria shows loose collagen fiber under the SLP **(A)** with distant fenestrations (arrows). The mid-region is arranged in an intertwined framework with frequent and large fenestration (**B**). Transition zones allow secure the vocal ligament **(C)**. Deep within the LP collagen fibers are densely packed and run parallel to the vocal fold edge **(D)**. Scalebar: 50 μm.

**Figure 7:**
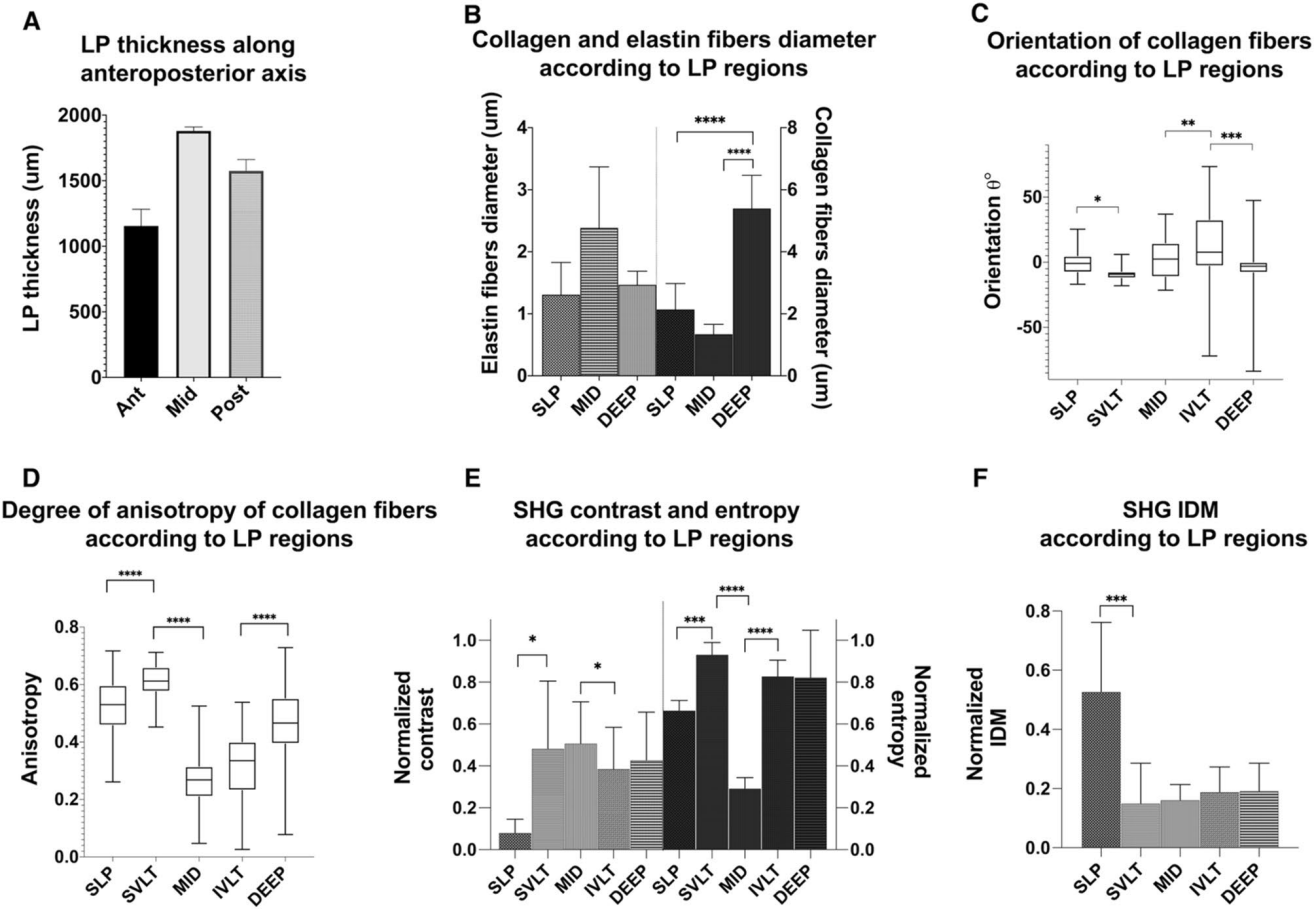
Quantitative analysis of SHG vocal fold features. The thickest section of the vocal fold (VF) along the anteroposterior axis **(A)** is in the mid-membranous region with 1.88 mm (± 0.02 mm). Collagen fibers are thicker than elastin fibers regardless of the lamina propria (LP) depth **(B)**. They are predominately aligned with the VF edge in the SLP and deep, as shown by the orientation **(C)** and degree of anisotropy **(D)** box plot. The second-degree statistics analysis shows that contrast and entropy **(E)** along with the inverse difference moment are useful features to distinguish the SLP from the superficial vocal ligament transition (SVLT).

Along its length, the VF ECM thickness depend on the anteroposterior location. It reaches a minimum thickness of 1.15 mm (± 0.13 mm) in the anterior region while the mid-membranous region reaches a peak depth of 1.88 mm (± 0.02 mm) (Fig. 7A) which is in line with previous histological investigation^81^. Figure 6 shows the mid-membranous ECM collagen distribution along its entire thickness from the SLP (Fig. 6A) to the deep region (Fig. 6D) of the LP. Thin longitudinal collagen fibers (2.1 ± 0.8 μm), mostly parallel to the VF edge, with few fenestrations are found in the SLP (Fig. 6A, pointed by arrows). At mid-depth, collagen fibers are superposed as a 3D lacework with the numerous large fenestrations (37% of the volume) (Fig. 6B). Inferiorly to this lattice-framework (Fig. 6C), collagen fibers are branching out in various directions to connect the deep layer, where collagen fiber bundles are thicker (5.4 ± 0.4 μm), tightly packed, and are mostly running parallel again to VF edge (Fig. 6D). A pairwise comparison of collagen fibers' diameter shows a significantly larger diameter in the deep LP compared to the SLP and MID (Fig. 7B). Although the MID and IVLT have the most spread angle distribution of collagen fibers, the most significant shift in fibers orientation is between the IVLT and DEEP (Fig. 7C). The highly organized SLP and SVLT structure is highlighted by the degree of anisotropy which is found to significantly differ based on the LP depth for all transitions, except for MID to IVLT, where no significant difference was found (P = 0.1632) (Fig. 7D). Although the MID and IVLT layers have collagen fibers running in different preferential orientations θ (a large standard deviation), the MID layer shows very organized collagen mesh with numerous fenestrations (void region). This regular symmetrical pattern, unique to the MID layer, is likely to increase the isotropy and therefore explains why this layer has the lowest anisotropy in the ECM. Table 1 summarize the quantitative morphometric evaluation of the human vocal fold collagen distribution using SHG imaging. The density (packed collagen fiber bundles) and the fenestration (number of void regions) of each LP segment are compared relatively to the region with the maximum density (DEEP, ++++) and fenestration (MID, ++++), respectively.

**Table 1:** Quantitative morphometric evaluation of the human vocal fold collagen distribution using SHG imaging.

LP region	Thickness (μm)	Fiber OD (μm)	Orientation (deg.)	Anisotropy	Fenestrations	Density
SLP	193.2 ± 11.3	2.1 ± 0.8	− 0.83 ± 9.1	0.52 ± 0.1	++	++
SVLT	158.8 ± 14.1	3.2 ± 1	− 9.5 ± 4.4	0.62 ± 0.06	+	+++
MID	236.6 ± 10.7	1.3 ± 0.3	2.9 ± 15	0.26 ± 0.09	++++	+
IVLT	242.1 ± 32.2	3.9 ± 1.5	13.5 ± 27.8	0.31 ± 0.11	+	+++
DEEP	635 ± 20.4	5.4 ± 0.4	− 5 ± 15.2	0.47 ± 0.12	+	++++

The correlation between the LP depth and the GLCM-based features such as normalized contrast, entropy, and inverse difference moment was evaluated on SHG images. All directions were extracted and averaged to account for all possible fiber orientations. The SLP has the lowest contrast (Fig. 7E) while the remaining LP shows a low homogeneity (Fig. 7F). This outcome is anticipated as fibers are aligned with the VF edge following a regular pattern in the SLP. The entropy is found minimal in the mid-region of the LP, which is directly correlated to a collagen density decline in that region. Transitions between SLP and the SVLT are significant for the contrast (P = 0.019), and extremely significant for entropy (P = 0.0003) and IDM (P = 0.0001). The significant difference between the same features evaluated at different depths of the extracellular matrix highlights morphological transitions in elastin and collagenous fibers' disposition.

### OCT imaging

#### ECM quantitative analysis

Although the images were similar between the probe and the commercial objective, the statistical analysis on the extracted features was performed using the in vivo contact probe data. The inherent in vivo imaging challenges (endoscopic measurements, SNR, field-of-view) have motivated this choice and convey a better clinical translation of those features.

**Figure 8:**
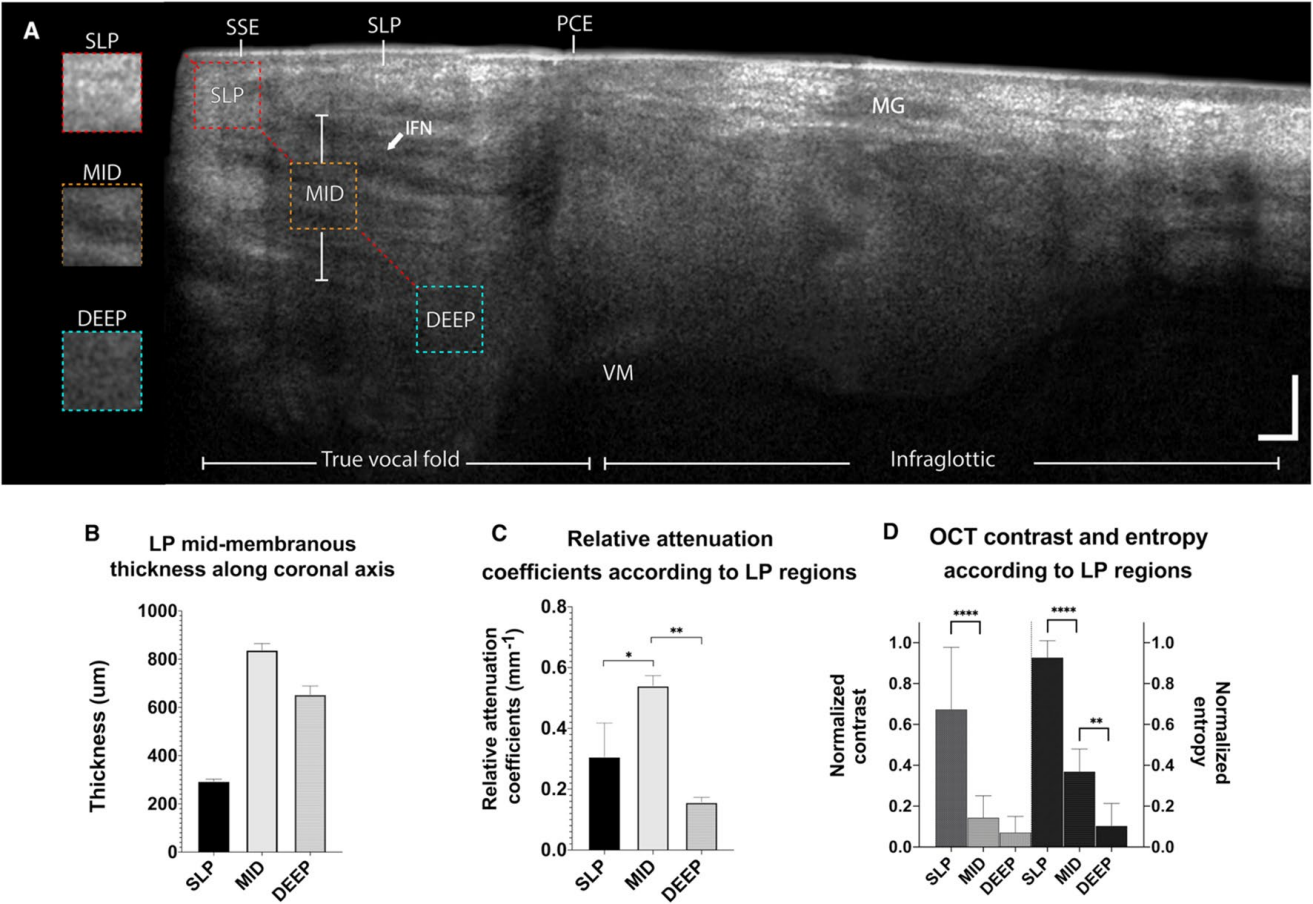
50-year-old female mid-membranous vocal fold. OCT cross-section with quantitative analysis of OCT features. The epithelium is clearly delineated from the lamina propria (LP) under the true vocal fold and infraglottic. The infraglottic is characterized by thickening to the epithelium and by the presence of numerous seromucous glands. Underneath the superficial LP is highly scattering. Under the SLP, near the edge, a darker textured region is visible, which corresponds to the intertwined fiber network (IFN arrow) noticeable under multiphoton imaging. The deep LP shows a random speckle typical of an attenuated signal. Insets are representing the texture of each LP section in depth **(A)**. The thickness along the coronal axis was extracted **(B)** and relative attenuation coefficients **(C)**. The normalized texture contrast and entropy from the GLCM were extracted along the 45°. midline (dash-line) **(D)**. *SSE* stratified squamous epithelium; *PCE* pseudostratified ciliated epithelium; *IFN* intertwined fiber network; *SLP* superficial LP; *MID* mid LP; *Deep* deep LP; *MG* seromucous glands; *VM* vocalis muscle. Scale bars: 500 μm.

Figure 8 shows an OCT cross-section captured with the endoscopic probe on the mid-membranous true VF of an intact 50-year-old female larynx. The epithelium appears as a uniform thin backscattering layer with a slight thickening in the infraglottic space suggesting a transition from squamous stratified epithelial cells (SSE) to a pseudostratified ciliated epithelium (PCE) layer. According to OCT textured images, underneath the epithelium, the LP can be segmented in superficial (SLP), mid (MID), and deep (DEEP). The SLP is clearly highlighted by a bright region (highly scattering). Beneath the SLP, more noticeable near the edge, an intensity variation characterizes the mid-section. This region correlates with the fiber intertwined network (IFN) observed under multiphoton imaging, which is around 250 μm below the basement membrane. The deep LP shows a uniform and smooth texture. Although the vocalis muscle structures are not visible under OCT, the steep absorption suggests the LP-VM interface.

#### ECM quantitative analysis

From mid-membranous OCT images along the 45-degree mid-line (dashed-line Fig. 8), the LP thickness and attenuation coefficients were extracted. The SLP thickness is 293.3 μm (± 9.3 μm), while the MID and DEEP sections are 837.6 μm (± 26.9 μm), and 653.2 μm (± 35.4 μm) thick, respectively. The corresponding relative attenuation coefficients ${u}_{t}$ for the SLP, mid and deep are 0.31 mm^−1^ (± 0.11 mm^−1^), 0.54 mm^−1^ (± 0.03 mm^−1^), and 0.15 mm^−1^ (± 0.01 mm^−1^), respectively. The LP depth was found to have a significant effect on the attenuation coefficient between the SLP and MID (P = 0.013) and with the MID and DEEP region (P = 0.001). The MID has the largest attenuation coefficient, which translates into a sharp exponential decline of the light. The contrast and entropy in the superficial layer are the highest, which is likely linked to the natural exponential signal decay. The quantitative morphometric evaluation of the human VF LP using OCT is summarize in Table 2.

**Table 2:** Quantitative morphometric evaluation of the human vocal fold lamina propria using OCT.

LP region	Thickness (μm)	${u}_{t}$(mm^−1^)
SLP	293.3 ± 9.3	0.31 ± 0.11
MID	837.6 ± 26.9	0.54 ± 0.03
DEEP	653.2 ± 35.4	0.15 ± 0.01

## Discussion

Quantifying the morphology of the vocal folds’ (VFs) biological structure has been the focus of numerous investigations over the last 40 years. However, our ability to evaluate VF extracellular matrix (ECM) homeostasis and the pathogenesis of benign and malignant lesions is still very limited. Since the distribution and arrangement of fibrous macromolecules are tightly tied to the VF biomechanical abilities^81^ and diseases^82^ , it is crucial to visualize and assess its microanatomy. The three-dimensional nature of the VFs suggests that non-invasive optical imaging techniques may be more suited to achieve this endeavor. By imaging the human larynx with multiphoton microscopy and optical coherence tomography (OCT) and then closely correlating this to anteroposterior stained histologic sections, we have aimed to address open questions (stated in the introduction) regarding the VF ECM microanatomy with the intent of finding optical features to guide phonosurgeries. The data acquired with label-free imaging on the full-length of the VF suggest that:

1. The VF LP microarchitecture is not a strict discrete three-layer structure but instead, a continuous 3D interwoven fiber matrix with different fibrillar arrangements as a function of ECM depth. As demonstrated, the different spatial arrangements of entangled elastic and collagen fibers are anchored with a predominant collagen mesh. This may perhaps be explained by damping transition zones between layers of different viscoelastic properties. This hypothesis is supported by studies that highlight the remodeling of the ECM (an increase of collagen fibril density) to respond to longitudinal and transverse strains^83^.
2. Although previous studies have shown that collagen fibers are running parallel along the VF free edge^9,47^, in our study, this was only observed within the SLP and, to some extent, deep in the LP.
3. In addition, the anterior LP was found to be thinner and overlaid by ciliated pseudostratified columnar cells, which is in line with previous histological investigations^84,85^. Those characteristics inherently contribute to different viscoelastic properties from the remaining two-thirds of the VF, thus explaining why mechanical, repetitive contact trauma or vocal misuse causes pathology in the anterior aspect of the VFs, such as with nodules, polyps, and cysts lesions. This hypothesis may be supported by some stress–strain test executed on the VF, where the stiffness near the anterior commissure was found to be higher than the mid-membranous portion of the VF^86^.
4. The quantitative morphometric evaluation of the human vocal fold lamina propria using optical features shows the potential to discriminate regions of the LP noninvasively. The various distributions in fibers diameter, orientation angles, and degree of anisotropy found in the ECM highlight the fibrous network's complexity. While our SLP findings correlate with those of Miri et al.^47^, we instead find an increase in the SHG signal intensity (collagen) in the deep region of the LP, which is an agreement with histochemical studies^87–89^. Similarly to a previous in vivo study^38^, we found that relative attenuation coefficients are good indicators of LP transitions in OCT. However, these features remain sensitive to noise, motion artifacts, and anatomical position. As the development of the vocal fold lamina propria is still unclear (age and gender), a selection away from the edge may be more susceptible to uncertainty in the anatomic position. The 45-degree mid-line landmark methodology developed in this study may remove selection bias and standardized quantitative evaluation of the VF morphology. Second-order statistics such as contrast and entropy indicated where sharp textures and local homogeneity are in the LP. These features are found to correlate well with oriented collagen fibers. However, the local uniformity (described by the inverse difference moment) was only significant between the SLP and the mid-region of the LP. Although the thickness distributions are the same, there is an offset of about 230 μm between OCT and multiphoton measurements. This difference encountered in the total LP depth is interpreted as an effect of shrinkage caused by the dehydration of the sample, as previously reported^90^.

A detailed map of the microanatomy through optical imaging could be indispensable to phonosurgeries. To this end, we mapped the relative composition of the VF with multiphoton imaging and OCT according to regions of the lamina propria. It is clear that depth and anteroposterior position dictate the density, arrangement, and maturity of elastic and collagen fibers. The spatial organization of elastin and collagen and different degrees of fenestration (space between fibers) may be of fundamental importance to the VF biomechanics. Knowing the fiber arrangement and approximately where those transitions occur may allow fine-tuning therapies and account for individual anatomical variability. It is notable that sparser fibrous element, visible in multiphoton microscopy, appears vibrant and highly scattering under OCT imaging, which is characteristic of numerous small scatters in a viscous medium. Beneath the SLP light scatters weakly in OCT, which correlates with the weak SHG signal (collagen) in the intertwined framework region. This confirms our previous study, where collagen concentration in the VF was found to be a significant source of optical scattering in OCT^74^.

This study comes with certain limitations that we acknowledge. Foremost, the number of specimens in this study is limited and does not highlight differences between age and gender, as previously reported by histological studies^6,91,92^. Although OCT acquisitions were performed on intact dissected larynges, multiphoton imaging acquisitions were acquired on cross-sections, subject to dehydration and fixation artifacts that inevitably affect the morphological and mechanical features of the VF. A possible solution would be to image the VF without sectioning. However, this would limit the penetration depth to 250–400 μm but should still be sufficient to access the SLP and perhaps the superior transition of the vocal ligament. Furthermore, our study does not differentiate between collagen fiber types (type I and III-VI) present in the LP, which may be helpful to expose lesions or and post-scar formation. Ongoing investigations are evaluating extracting collagen fiber types through a susceptibility tensor analysis with a polarization-resolved SHG microscope^93,94^ and combining outcomes to a birefringence analysis using polarization-sensitive OCT^95,96^. We anticipate that the spatial arrangement of entangled collagen fibers will generate a different gradient of birefringence. The system sensitivity in OCT (roll-off effect) is an important factor in estimating the attenuation coefficients. The system used in this study (VCSEL) has a long coherence length and can maintain its sensitivity at > 100 dB over a large imaging depth^97^. Considering that our imaging window is within 2 mm, the effect of roll-off was neglected here. However, as previously reported^98^, a calibration tool would be a valuable adjunct to improve data analysis and longitudinal or multicenter comparison.

Nevertheless, despite the small sample size, it is apparent that predicting surgical outcomes and optimizing voice therapy is contingent on a proper evaluation of the microscopic LP features. It would be interesting to combine relevant pixel-based, A-Line-based, and layer-based features with a machine learning classifier for automatic recognition of the LP regions. This study reports the first quantitative assessment of the entire vocal fold ECM using multiphoton and OCT. Future biomechanical investigations may take advantage of those parameters to characterize and quantify the impact of stain-stiffness experiments on collagen fibers at global and local levels in real-time. Furthermore, as pathophysiology plays a role in the remodeling of the lamina propria, those quantitative values and features may help understand the etiology of some benign and malignant lesions.

## Conclusion

Optical imaging can assess the VF under various physiological and pathological conditions heretofore unexplained, which cannot be accomplished with traditional imaging techniques such as ultrasound, MRI, and CT. It also provides a novel means of tracking human development and linking diagnostics with therapeutic imaging modalities to treat pathologies such as dysplasia and frank neoplasia. This study focused on the optical contrast of VF ECM with an eye toward real-time phonosurgery guidance. A full assessment of the VF ECM macromolecule composition and fiber distribution was performed with TPEF, SHG, OCT, and further validated with histopathology. We demonstrated that the lamina propria, the shock absorber of the VF, is markedly thinned in the anterior one-third of the VF itself and may account for the propensity of the development of some laryngeal diseases in this region. We further examined and extracted the relationship between OCT and multiphoton VF imaging, promoting correspondences that could lead to accurate 3D mapping of the VF architecture in real-time during phonosurgeries. However, further investigations are required, such as performing real-time imaging while applying a deformation on the laryngeal structure, mimicking stress involved during phonation.
