# Publisher Correction: Quantitative evaluation of the human vocal fold extracellular matrix using multiphoton microscopy and optical coherence tomography

**DOI:** 10.1038/s41598-021-84391-7

**Published:** 2021-02-22

**Authors:** Fouzi Benboujja, Christopher Hartnick

**Affiliations:** Department of Otolaryngology, Massachusetts Eye and Ear Infirmary, Harvard Medical School, 243 Charles Street, Boston, MA 02114 USA

Correction to: *Scientific Reports* 10.1038/s41598-021-82157-9, published online 28 January 2021

The original version of this Article contained a typographical error in the spelling of the author Christopher Hartnick, which was incorrectly given as Chri stopher Hartnick.

Furthermore, this Article contained a typographical error in Equation 4,

$$\langle I\left(z\right)\rangle \propto {I}_{0}\mathrm{ exp}(-2{u}_{t}\cdot z$$

now reads:

$$\langle I(z)\rangle \propto {I}_{0}\mathrm{ exp}(-2{u}_{t}\cdot z)$$

These errors have now been corrected in the PDF and HTML versions of the Article.

